# Involvement of *DkTGA1* Transcription Factor in Anaerobic Response Leading to Persimmon Fruit Postharvest De-Astringency

**DOI:** 10.1371/journal.pone.0155916

**Published:** 2016-05-19

**Authors:** Qing-gang Zhu, Miao-miao Wang, Zi-yuan Gong, Fang Fang, Ning-jing Sun, Xian Li, Donald Grierson, Xue-ren Yin, Kun-song Chen

**Affiliations:** 1 Zhejiang Provincial Key Laboratory of Horticultural Plant Integrative Biology, Zhejiang University, Zijingang Campus, Hangzhou, PR China; 2 The State Agriculture Ministry Laboratory of Horticultural Plant Growth, Development and Quality Improvement, Zhejiang University, Zijingang Campus, Hangzhou, PR China; 3 Department of Horticultural Sciences, College of Agriculture, Guangxi University, Nanning, PR China; 4 Plant & Crop Sciences Division, School of Biosciences, University of Nottingham, Sutton Bonington Campus, Loughborough, United Kingdom; South China Agricultural University, CHINA

## Abstract

Persimmon fruit are unique in accumulating proanthocyanidins (tannins) during development, which cause astringency in mature fruit. In ‘Mopanshi’ persimmon, astringency can be removed by treatment with 95% CO_2_, which increases the concentrations of ethanol and acetaldehyde by glycolysis, and precipitates the soluble tannin. A TGA transcription factor, *DkTGA1*, belonging to the bZIP super family, was isolated from an RNA-seq database and real-time quantitative PCR indicated that *DkTGA1* was up-regulated by CO_2_ treatment, in concert with the removal of astringency from persimmon fruit. Dual-luciferase assay revealed that *DkTGA1* had a small (less than 2-fold), but significant effect on the promoters of de-astringency-related genes *DkADH1*, *DkPDC2* and *DkPDC3*, which encode enzymes catalyzing formation of acetaldehyde and ethanol. A combination of *DkTGA1* and a second transcription factor, *DkERF9*, shown previously to be related to de-astringency, showed additive effects on the activation of the *DkPDC2* promoter. Yeast one-hybrid assay showed that DkERF9, but not DkTGA1, could bind to the *DkPDC2* promoter. Thus, although *DkTGA1* expression is positively associated with persimmon fruit de-astringency, trans-activation analyses with *DkPDC2* indicates it is likely to act by binding indirectly *DkPDC2* promoter, might with helps of DkERF9.

## Introduction

Persimmon (*Diospyros kaki* L.) is a crop of high economic importance in China. There are two main types, either non-astringent or astringent [1]. However, most persimmon fruit have astringency at the commercial mature stage [2], caused by accumulation of soluble condensed tannins (proanthocyanidins, SCTs). Artificial postharvest treatments have been developed to remove astringency, including high CO_2_ (usually >90%), ethylene and ethanol [3–7]. CO_2_ is the most effective treatment and depends on the production of acetaldehyde [6,8,9].

The biochemical basis of postharvest de-astringency in persimmon has been comprehensively investigated. The activities of pyruvate decarboxylase (PDC, EC 4.1.1.1) and alcohol dehydrogenase (ADH, EC 1.1.1.1) have been shown to increase during CO_2_ treatment [6,10,11] leading to the formation of acetaldehyde and ethanol from pyruvate [12–14] and the acetaldehyde produced precipitates the soluble tannins. More recently, the genes involved in this process have been investigated. Five *PDC* genes and three *ADH* genes have been isolated from persimmon and their expression shown to be induced by the application of CO_2_ [6]. Transient over-expression of *DkPDC2* rapidly decreased SCT in persimmon leaves [6], thus, *DkPDC2* is an important structural gene for persimmon fruit de-astringency. Five transcription factors were reported to be involved in persimmon de-astringency regulation, including four ethylene responsive factor genes (*DkERF9/10/19/22*) and a MYB transcription factor (*DkMYB6*) [6,7,15]. All five transcription factor could transcriptionally activated de-astringency related target genes, meanwhile *DkMYB6* could also enhance activity of the *DkERF19* promoter, which suggested possible transcriptional regulatory cascades involving different transcription factors. Thus, de-astringency is based on the induction under anaerobic conditions of genes leading to acetaldehyde production, but more information about the transcriptional regulation of these processes is still limited.

In *Arabidopsis*, at least four *ERF* genes, including *HRE1*, *HRE2*, *RAP2*.*2* and *RAP2*.*12* have been identified as the main plant oxygen-sensing regulators. HRE1 and RAP2.2 could transcriptionally regulate the anaerobic fermentation-related *ADH* and *PDC* genes [16,17], and lines containing T-DNA knockouts of *RAP2*.*2* had lower survival rates than the wild type in *Arabidopsis* [16]. Moreover, some other transcription factors have also been reported as hypoxia responsive, such as *AtMYB2*, which is induced by hypoxia, can bind to the promoter of *AtADH1* [18], and increased the expression of *AtADH1* when overexpressed [19]. Also, in wheat, *TaMYB1*has been shown to be responsive to low oxygen [20] and recent omics-based studies have highlighted more hypoxia responsive genes, and RNA-seq has been used to identify more than 180 transcription factors differentially expressed in response to low oxygen [21]. Thus, it is likely that more transcription factors would be expected to be involved in persimmon fruit postharvest de-astringency.

In the present research, a TGA-like transcription factor, *DkTGA1*, was identified, using data from the RNA-seq analysis performed by Min et al. [7]. The expression pattern of *DkTGA1* in response to the application of 95% CO_2_ was studied by real-time PCR. The potential role(s) of *DkTGA1* in regulating previously characterized deastringency-related target genes were investigated, using dual-luciferase assay, testing synergistic interactions between *DkTGA1* and *DkERF* genes and yeast one hybrid analysis.

## Materials and Methods

### Plant materials and treatment

Commercial mature persimmon (*Diospyros kaki* L. cv. Mopanshi) fruit were harvested and bought from a commercial orchard (Yangjingliang’s farm) at Fangshan (Beijing, China) in 2010. We confirm that the field studies did not involve endangered or protected species. Fruit were transported to the laboratory at Zhejiang University (Hangzhou, China) and then were selected with uniform size, free from visible mechanical wounds for postharvest treatments. Selected fruit were divided into two batches, one treated with 95% CO_2_ for the de-astringency and the other (control) placed in air, with both batches held in air-tight plastic containers for 1 d. After treatments, the fruit were held in air at 20°C for 4 d. All of the treatments were performed with three biological replicates. At each sampling point, fruit flesh samples were separately taken from three replicates of four fruit each. The samples were frozen in liquid nitrogen and stored at -80°C until further use.

### Fruit physiology evaluation

In order to reflect fruit astringency, SCTs were measured using Folin-Ciocalteu reagent (Sigma) according to the method described by Yin et al. [5]. The results were expressed as tannin acids equivalents per g^-1^ fresh weight.

Acetaldehyde and ethanol production were measured with a gas chromatograph (Agilent 6890N, USA), fitted with a FID column (HP-INNOWAX, 0.25mm, 30m, 0.25μm, Agilent J&W, CA, USA). Measurements were conducted according to our previous report [6]. In brief, 2 g frozen flesh sample was ground in liquid nitrogen and added to 5 ml saturated NaCl. Three ml of the mixture were transferred to 10 ml air-tight vials with crimp-top caps, and were incubated at 60°C in a water-bath for 1 h. Then, 0.2 ml head-space gas was removed for gas chromatography. The injector, detector and oven temperatures were set at 150, 160 and 100°C, respectively. Sec-butyl alcohol (Sigma) was used as an internal standard. The results were calculated using standard curves for acetaldehyde and ethanol, respectively.

### RNA extraction and cDNA synthesis

Total RNA was extracted from frozen fruit flesh (2.0 g) by the method developed by Chang et al. [22]. The trace amount of genomic DNA in total RNA was digested with TURBO DNA free kit (Ambion). First strand cDNA was synthesized from 1.0 μg DNA-free RNA, using iScript cDNA Synthesis Kit (Bio-Rad). For each sampling point, three biological replicates were used for RNA extraction and subsequent cDNA synthesis.

### Gene Isolation and Sequence Analysis

RNA-seq analysis was conducted with CO_2_-treated persimmon fruit, which were described in Min et al. [7]. A TGA-like de-astringency responsive unigene was indicated by RNA-seq. The full-length *TGA* gene was isolated with a SMART RACE cDNA Amplification Kit (Clontech). The sequences of primers used for RACE and full-length amplification of *DkTGA1* are described in Table 1. Based on the deduced amino acid sequences, a phylogenetic tree of *TGA* genes was generated using Clustal X (v 1.81) and MEGA (v 6.0). The deduced of amino acid sequences of homologous genes in *Arabidopsis* were obtained from NCBI.

**Table 1:** Sequences of the primers used for RACE, full-length amplification, real time PCR and vector construction

Genes	Methods used	Primers (5’-3’)
*DkTGA1*	3’RACE (Primary PCR)	CAACAGGGTCTGTATATAGGTGGTGG
*DkTGA1*	3’RACE (Secondary PCR)	GGTCTGGATAAACTCCAGCAAACT
*DkTGA1*	5’RACE (Primary PCR)	GTAAGAGCATCTTCCAATTGCTGACA
*DkTGA1*	5’RACE (Secondary PCR)	TTGGTCAAACTTTTTTGAAGGTAGT
*DkTGA1*	Full-length clone (FP)	CTCAAAGGTCATATTGGAAACTCA
*DkTGA1*	Full-length clone (RP)	AGTTCCATCTAAAGGCACTCT
*DkTGA1*	Q-PCR (FP)	TCATCTTCGCAAGGAAACGC
*DkTGA1*	Q-PCR (RP)	CGACTAGGCAGGCTCGTGAA
*DkTGA1*	SK vector construction (FP)	CGCGTCGACATGACCTCTCCAACTGC
*DkTGA1*	SK vector construction (RP)	GACGGTACCCTAGGCAGGCTCGTGA
*DkTGA1*	Y1H constructs (FP)	CGCCATATGATGACCTCTCCAACTGC
*DkTGA1*	Y1H constructs (RP)	GACGGATCCCTAGGCAGGCTCGTGA
*DkERF9*	Y1H constructs (FP)	CATGAATTCATGGTTGGCTTTGTGAA
*DkERF9*	Y1H constructs (RP)	GCAGGATCCTCATCCAGCAGCTTCA

Note: underlined sequences show cutting sites for restriction enzymes.

### Real-time PCR analysis

Oligonucleotide primers for real-time PCR analysis were designed with primer premier 5.0. The specificity of primers was determined by melting curves and PCR products resequencing. The sequences of oligonucleotide primers are in Table 1. Primers used for *DkADH*, *DkPDC* and *DkERF* genes were the same as in previous reports [7].

Real-time PCR was carried out with LightCycler 480 SYBR Green I Master (Roche) and LightCycler 480 II instrument (Roche) for gene expression studies. The PCR reaction mixture (20 μl total volume) comprised 10 μl 2 × real-time PCR mix (Roche), 1 μl of each primer (10 μM), 2 μl diluted cDNA, and 6 μl Diethy pyrocarbonate (DEPC) H_2_O. The PCR program was initiated for 3 min at 95°C, followed by 50 cycles of 95°C for 10 s, 55°C for 30 s, and completed with a melting curve analysis program. The relative expression of each gene was calibrated with values for day 0 fruit set as 1. The housekeeping gene, *Actin*, was used as the internal control [1].

### Dual Luciferase assay

The trans-activation ability of *DkTGA1* on de-astringency related genes was investigated by dual luciferase assay. Full-length *DkTGA1* was inserted into pGreen II 002962-SK vector, using the primers listed in Table 1. Full-length *DkERF* and *DkADH* and *DkPDC* promoter constructs were obtained from our previous studies [6,7]. All constructs were electroporated into *Agrobacterium tumefaciens* GV3101. The dual luciferase assay was performed with *Nicotiana benthamiana* leaves, using the protocol described by Min et al. [6,7]. Dual luciferase assays were carried out in three independent experiments, with five biological replicates for each experiment.

### Yeast One-hybrid Assay (Y1H)

The yeast one-hybrid assays were performed, using the Matchmaker Gold Yeast One-hybrid Library Screening System (Clontech, USA). As described in Min et al. [7], *DkADH1* and *DkPDC3* showed auto-activation activities, thus only *DkPDC2* promoter was included for yeast one-hybrid analysis. The full-lengths of *DkERF9* and *DkTGA1* were subcloned into pGADT7 AD vector (AD) (primers are listed in Table 1). Transformed Y1HGold were cultured on SD/-Leu containing 200 ng/ml aureobasidin A at 30°C for 3 d to test interaction. pGADT7-Rec (AD-Rec-P53) was co-transformed with the p53-promoter fragment in Y1HGold as positive control.

### Statistical Analysis

Statistical significance of differences was calculated using least significant difference (LSD) with DPS software (v.3.11).

## Results

### Gene isolation and sequence analysis

**Fig 1:**
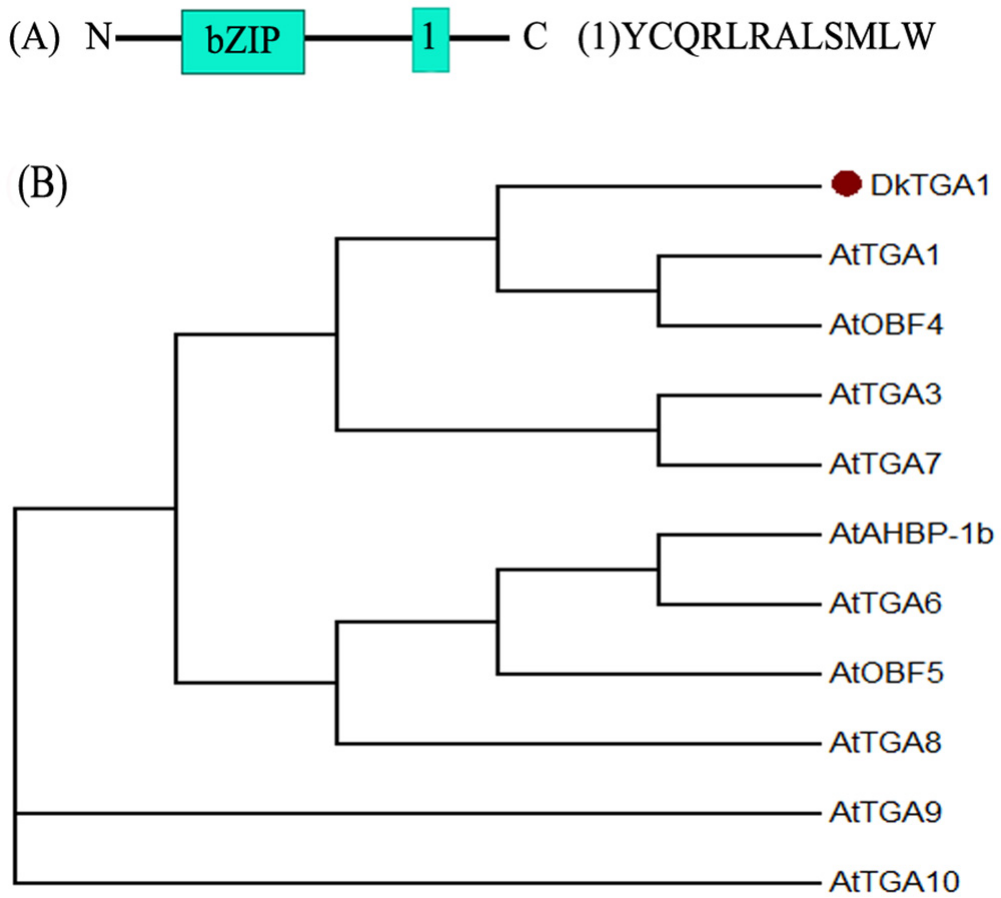
Phylogenetic tree of *TGA*. **(A) Schematic analysis of *DkTGA1***. The bZIP domain and TGA related motif are indicated in blue. (B) Persimmon *DkTGA* is indicated with a red dot. The amino acid sequences of AtTGA transcription factors were obtained from The Arabidopsis Information Resource or National Center for Biotechnology Information. The phylogenetic tree was constructed with MEGA (v 6.0)

One hypoxia responsive *TGA*-like unigene, which showed a 25-fold induction by CO_2_ treatment, was identified by RPKM (Reads per kb per million reads) from RNA-seq (data not shown) and the full-length sequence was obtained using RACE. Sequence analysis indicated that this differentially expressed gene had a YCQRLRALSMLW domain (Fig 1a), which is a characteristic of *TGA* genes [23], and was designated as *DkTGA1* (KU589287).

A phylogenetic tree was generated from the deduced amino acid sequences of the *DkTGA1* and 10 *TGA* genes from *Arabidopsis thaliana*, which have been divided into five clades [24,25]. *DkTGA1* showed the highest homology to clade I that comprises *AtTGA1* (At5g65210) and *AtTGA4* (At5g10030) (Fig 1b).

### Association between *DkTGA1* mRNA accumulation and persimmon fruit postharvest de-astringency

**Fig 2:**
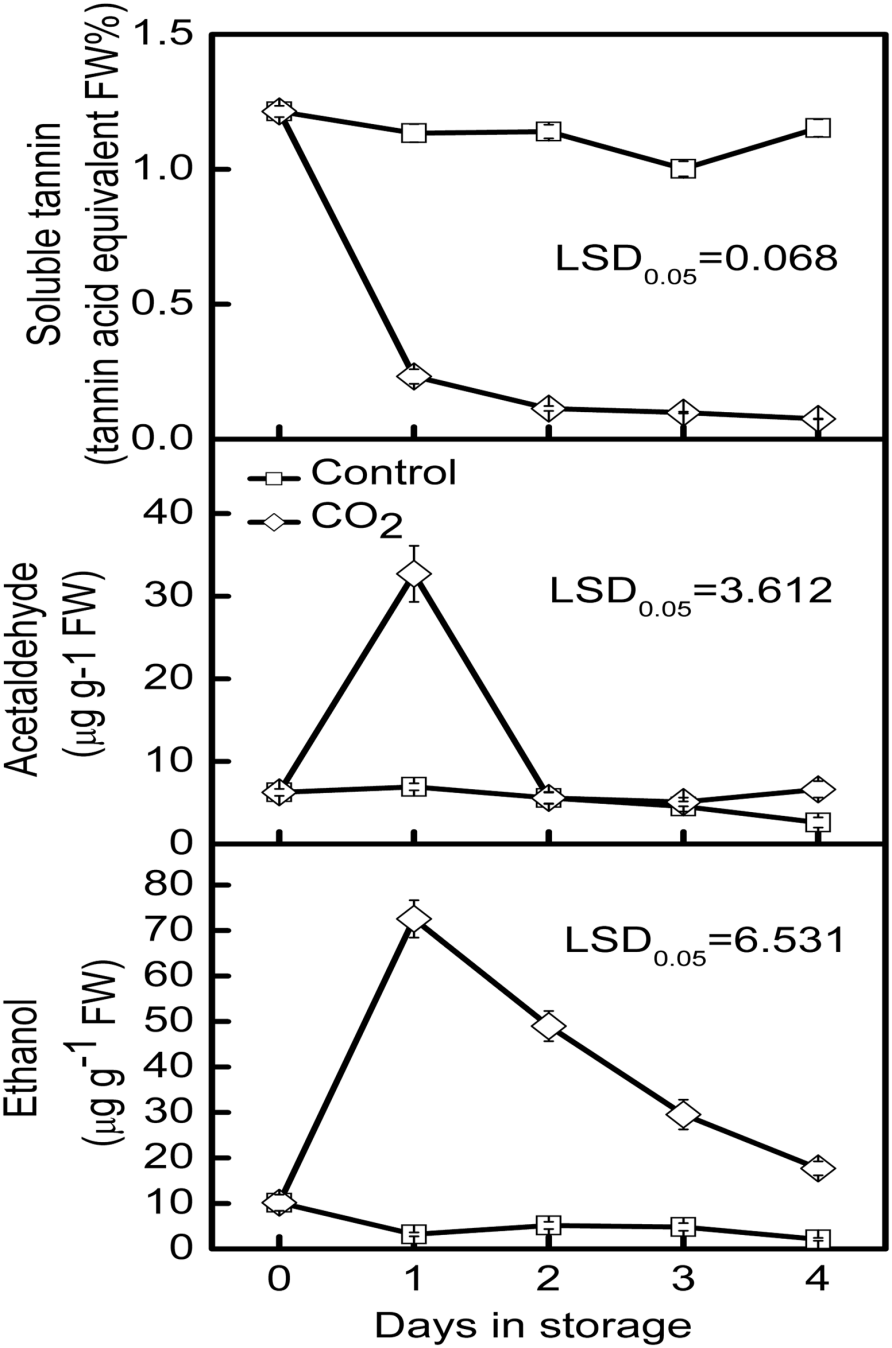
Changes of soluble tannins, acetaldehyde and ethanol in ‘Mopanshi’ persimmon fruit in response to treatment with 95% CO_2_ for 1 day. Error bars represent ±SE from three replicates. LSDs represent least significant difference at *p* = 0.05.

CO_2_ treatment (95%) promoted persimmon fruit de-astringency and caused a rapid decrease in the concentration of soluble tannin from 1.214% at 0 d to 0.232% at 1 d while in the control fruit the soluble tannin content was almost constant during storage (Fig 2). Accumulation of acetaldehyde and ethanol, the products of anaerobic respiration, were observed in CO_2_-treated fruit, concomitantly with loss of astringency-related SCT. Acetaldehyde increased from 6.26 μg/g at 0 d to 32.70 μg/g after CO_2_ treatment while the ethanol increased from 10.18 μg/g to 72.56 μg/g (Fig 2). Following removal of CO_2_ treatment, acetaldehyde content rapidly declined to the basal level observed at 0 d, while the decrease in ethanol content occurred more slowly than acetaldehyde, and remained higher than that in control fruit (Fig 2).

**Fig 3:**
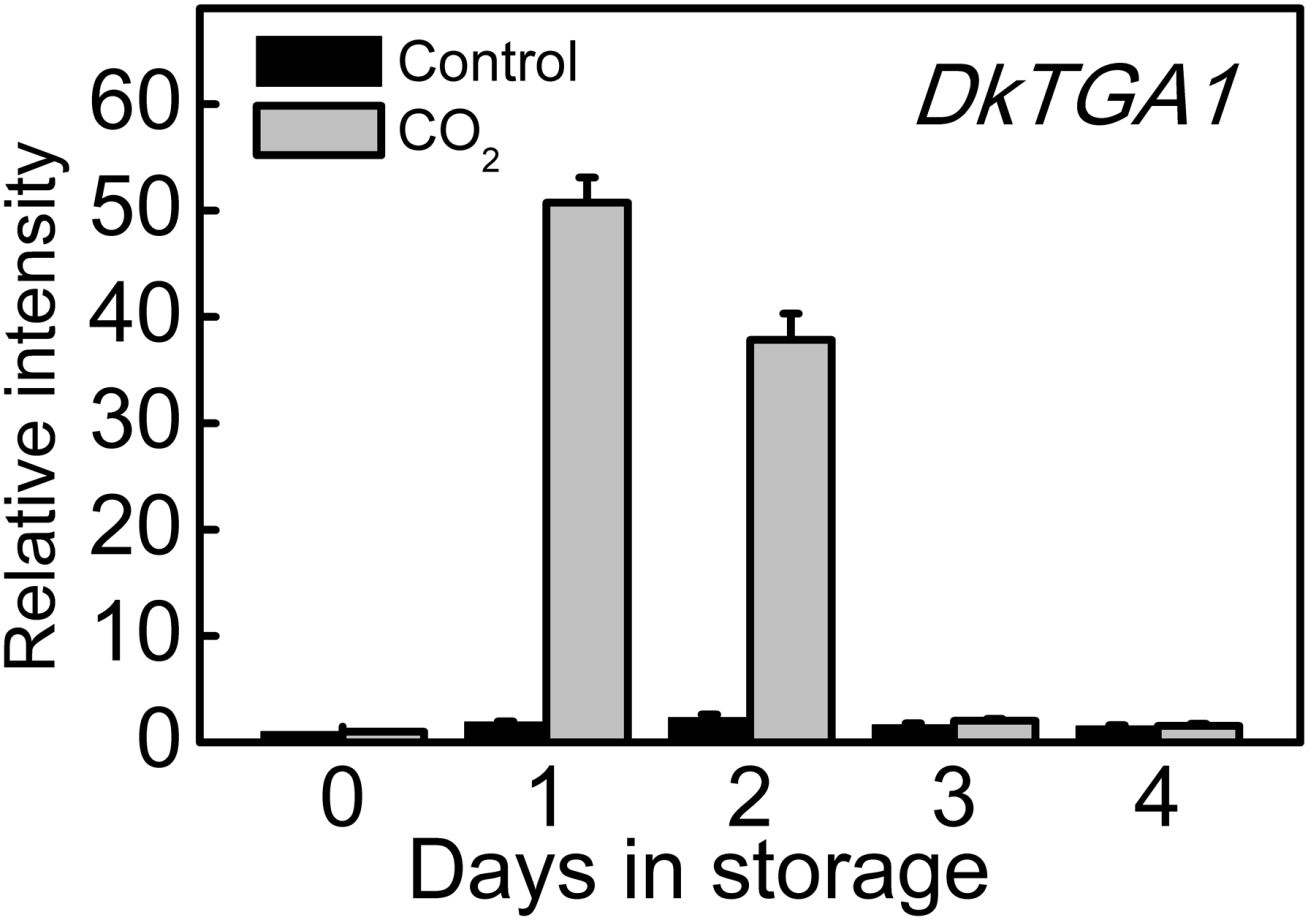
Expression of *DkTGA1* in response to CO_2_ treatment (95%, 1 day). Relative mRNA abundance was evaluated by real-time PCR. Day 0 fruit values were set as 1. Error bars represent ±SE from three replicates. LSDs represent least significant differences at *p* = 0.05.

Transcripts of *DkTGA1* was transiently induced during CO_2_ treatment, peaking after 1 d, with an approximate 50-fold induction at 1 d, and then decreased to similar level of that in control fruit following removal of CO_2_ (Fig 3). All of the previously isolated de-astringency related genes, *DkADH1*, *DkPDC2*, *DkPDC3*, *DkERF9*, *DkERF10*, *DkERF19* and *DkERF22*, were up-regulated by CO_2_ treatment (S1 Fig), which confirmed previous observations [6,7].

### Trans-activation of persimmon *DkADH1*, *DkPDC2* and *DkPDC3* promoters by DkTGA1

**Fig 4:**
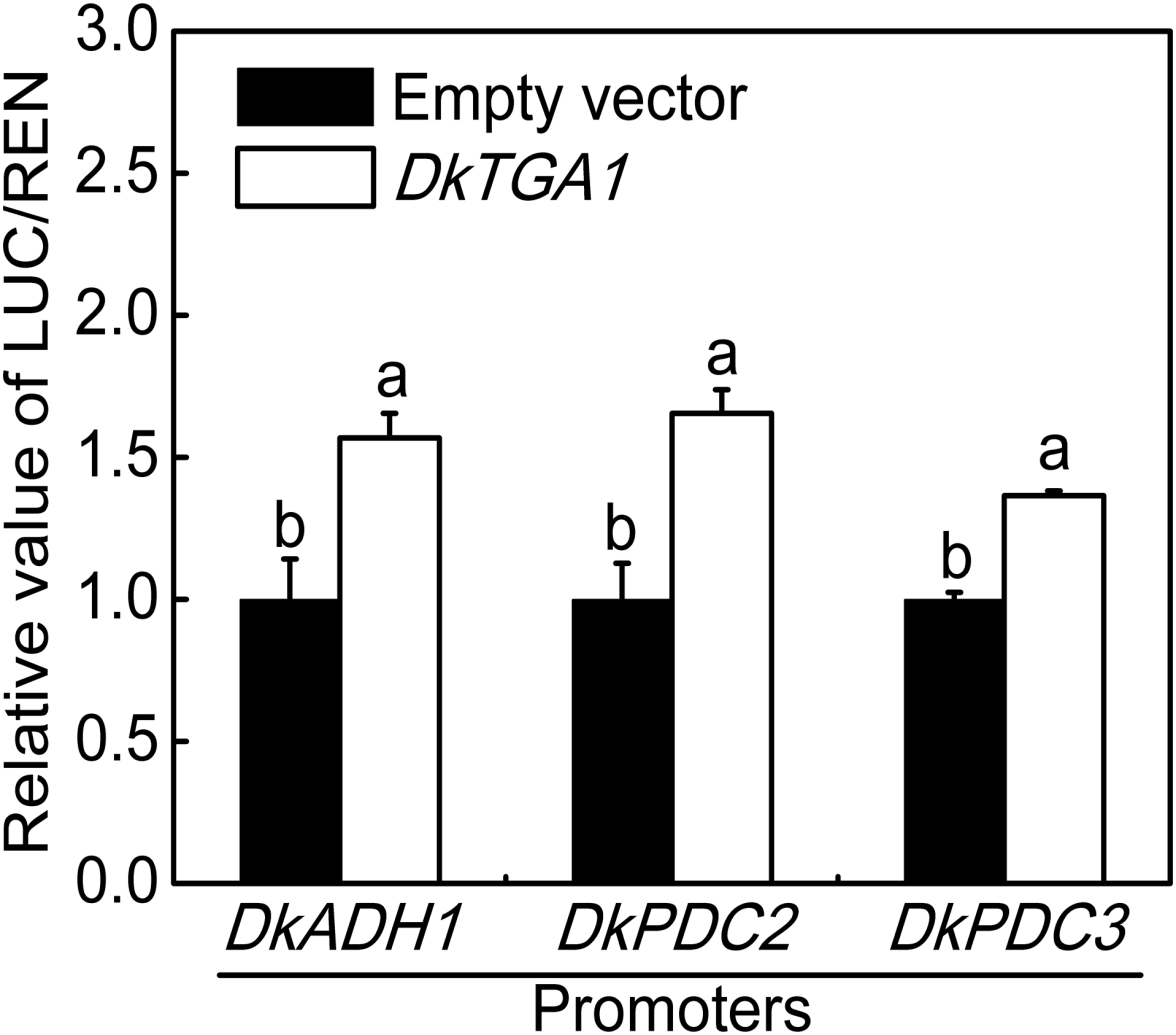
Trans-activation effects of *DkTGA1* on the promoters of deastringency-related genes (*DkADH1*, *DkPDC2*, *DkPDC3*) using the dual-luciferase assay. The value of LUC/REN for the empty vector (SK) was set as 1. Error bars indicate S.E.s from five replicates. Different letters above the columns indicate significant differences (*P* < 0.05)

A dual luciferase assay was carried out to investigate the possible transcriptional regulatory linkage between DkTGA1 and target genes known to be involved in astringency removal. The results indicated that *DkTGA1* had a small (but statistically significant) effect on genes induced by anaerobic conditions and involved in the de-astringency process, including *DkADH1*, *DkPDC2* and *DkPDC3*, with an approximately 1.5-fold activation of the promoters of these genes (Fig 4).

### Synergistic effect of *DkTGA1* and *DkERF* on trans-activation of promoters of de-astringency-related target genes

It was shown previously that DkERF9 and DkERF19 could activate the *DkPDC2* promoter while DkERF10 activated the *DkADH1* promoter [6,7]. As shown in Fig 4, DkTGA1 could trans-activate the *DkADH1*, *DkPDC2* and *DkPDC3* promoters. Thus, further investigations were conducted using *DkERF9*, *DkERF10* and *DkERF19* promoters, however, the results indicated that DkTGA1 also had very limited effects on promoters of these *DkERF* genes (S2 Fig).

**Fig 5:**
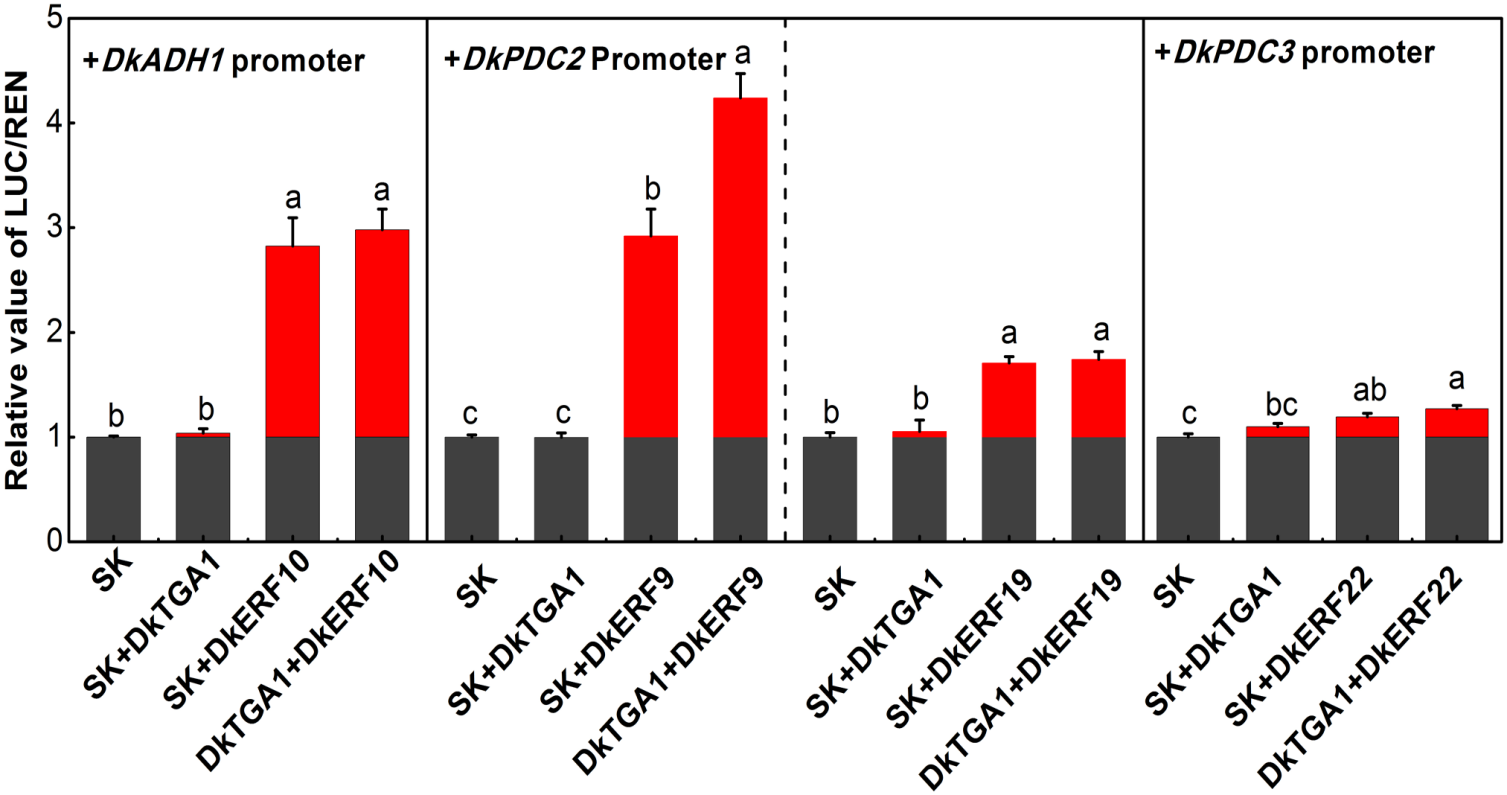
Synergistic effect of DkTGA1 and DkERF on trans-activation of the promoters of deastringency-related genes (*DkADH1*, *DkPDC2*, *DkPDC3*) using the dual-luciferase assay.

Subsequently, the effect of combining DkTGA1 and DkERF transcription factors was analyzed. DkTGA1 and DkERF9 together significantly enhanced promoter activation, compared with the effects of DkTGA1 or DkERF9 singly (Fig 5). However, no such enhancement was found when DkTGA1 was tested in combination with DkERF10, DkERF19 or DkERF22 (Fig 5).

**Fig 6:**
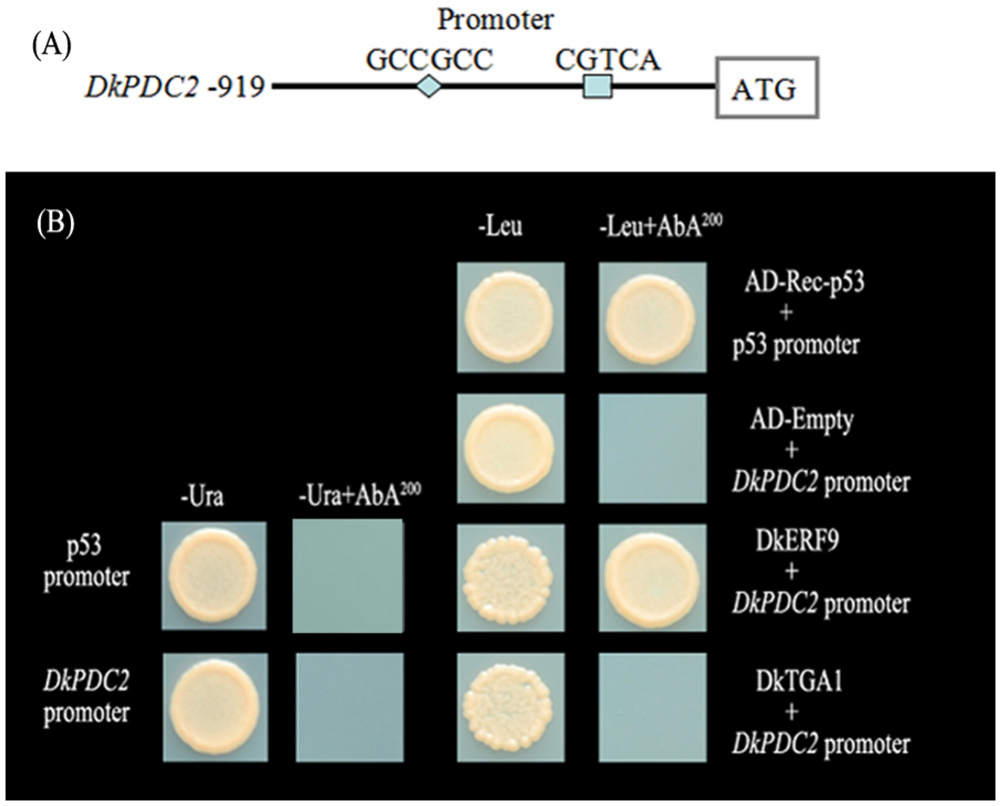
Yeast one-hybrid analysis of DkTGA1 and DkERF9 binding to promoter of *DkPDC2*. (A) Schematic representation of *DkERF* (GCCGCC) and *DkTGA* (CGTCA) possible binding sites in *DkPDC2* promoter. (B) Auto-activation of promoters were tested on SD medium lacking Ura in presence of aureobasidin A (AbA). Interaction was determined on SD medium lacking Leu in presence of AbA.

Yeast one/two-hybrid assays were performed in view of the results obtained from the dual luciferase assay. Motif analysis indicated the existing of the potential ERF and TGA binding sites (Fig 6A). Yeast one-hybrid results indicated that DkERF9 could bind to *DkPDC2* promoter, whereas no physical interaction was found between DkTGA1 and the *DkPDC2* promoter (Fig 6B). However, yeast two-hybrid studies indicated that there was no direct protein-protein interaction between DkTGA1 and DkERF9 (S3 Fig), despite the additive effects observed between DkTGA1 and DkERF9 on expression from the *DkPDC2* promoter.

## Discussion

Persimmon astringency removal had been widely studied and several technologies had been applied to reduce the SCTs in persimmon fruit, including treatment with high concentrations of CO_2_ or N_2_ [4,6,7,12,26], C_2_H_4_ treatment [5], dipping in hot water [27], all of which can lead to the persimmon fruit astringency removal. The effectiveness of CO_2_ treatment is widely considered to be due to the hypoxia response, which stimulates acetaldehyde production in persimmon fruit by anaerobic respiration, which precipitates the insoluble tannins, thereby removing astringency [6,15,28]. The results obtained in the present research are entirely consistent with this, as a rapid decrease in SCTs and transient stimulation of acetaldehyde and ethanol were observed in ‘Mopanshi’ persimmon in response to 95% CO_2_ treatment.

A few transcription factors have been suggested to be involved in the de-astringency response in persimmon fruit, however, only five transcription factors have been verified as having trans-activation ability on promoters of de-astringent related target genes (eg. *DkADH1*, *DkPDC2* and *DkPDC3*). These include four *DkERF* genes (*DkERF9/10/19/22*) [6,7] and a MYB transcription factor (*DkMYB6*) [15]. Here, a de-astringency responsive bZip transcription factor, detected in an RNA-seq database and designated as *DkTGA1*, based on phylogenetic comparisons with related genes from other plants. TGA is a member of a sub-class of bZIP transcription factors that have been widely reported to be involved in stress responses, for instance, *AtTGA1-7* has been characterized with respect to their interaction with *pathogenesis-related gene1* (*NPR1*) [29]; *AtTGA1* and *AtTGA4* also played important roles in Arabidopsis root developmental responses to nitrate [30]. *DkTGA1* was clustered with *AtTGA1* and *AtTGA4*, suggesting *DkTGA1* may also participate in response to stress in persimmon fruit. Although no TGA genes have been reported as being responsive to low oxygen in other plants, the present results indicate a significant (50-fold) induction of *DkTGA1* expression in response to high CO_2_/low O_2_ treatment (Fig 3). This suggests that the involvement of TGA genes in the anoxia response in other plants should be investigated. *DkTGA1* had a small transactivation effect on the promoters of *DkADH1*, *DkPDC2* and *DkPDC3*, which further indicated its possible involvement in persimmon de-astringency and the hypoxia response. However, the effects of *DkTGA1* on de-astringency related target genes were weaker than our previously reported results for other transcription factors such as *DkERF9/10/19/22* and *DkMYB6* [6,7,15]. It is notable that increasing expression of *DkTGA1* only occurred at 1 d and 2 d, which is similar to *DkERF9/10/19/22* [6,7] and differ to *DkMYB6* [15]. As deastringent process for ‘Mopanshi’ fruit in response to high CO_2_ treatment, generally happened on 1 d or 2 d, thus expression patterns of *DkTGA1*, as well as *DkERF9/10/19/22*, suggested that these transcription factors might be more specific for deastringency.

It is worth emphasizing that the synergistic effects of DkTGA1 and DkERF9 were observed on *DkPDC2* promoter. Synergistic effects of different transcription factors have been widely reported, for example MaNAC1 is involved in cold tolerance of banana fruits, and interacts with MaCBF1 [31] and EjAP2-1 interacts with EjMYB1/2 to form a complex involved in loquat fruit lignification [32]. Moreover, other TGAs and ERFs have been shown to interact with each other, for instance, AtTGA4 interacts with AtEBP (ethylene-responsive element binding protein, the previous name for *ERF*), which binds the ethylene response promoter elements and may therefore be important in regulating gene expression during the plant defense response [33]. However, additive effects of DkTGA1 and DkERF9 were not due to direct protein-protein interaction between, as Y2H experiments indicated no interaction between two transcription factors. Thus, synergistic effects of DkTGA1 and DkERF9 not only raised a possible involvement of a new transcription factor(s) in persimmon fruit de-astringency, but also provided a new evidence for relationships, with unknown mechanisms, between members of the TGA and ERF families, different from those previously reported. It is clear that DkERF9 can physically interact with the *DkPDC2* promoter, while *DkTGA1* is not able to bind directly to the promoter. These results suggest that *DkTGA1* is a novel and indirect regulator of persimmon fruit postharvest de-astringency, and may function via indirect interaction with DkERF9, a direct regulator on *DkPDC2*. As persimmon fruit deastringency related transcription factors are still limited, future researches might bridge the gap between *DkTGA1* and deastringency.

## Conclusion

In conclusion, CO_2_ (95%) treatment is effective in promoting and accelerating persimmon fruit postharvest de-astringency. The present study isolated a de-astringency inducible *DkTGA1*, which is an indirect activator of anaerobic response target genes. Furthermore, DkTGA1 and DkERF9 were shown to have an additive effect on activation of *DkPDC2*. As CO_2_-driven persimmon fruit postharvest de-astringency is mainly based on fermentation under anoxia, the activity of *DkTGA1* suggests the likely involvement of *TGA* transcription factors in hypoxia responses in other plants.

## Supporting Information

S1 Fig Expression of previously characterized deastringency related *DkERF*, *DkADH* and *DkPDC* genes in response to CO_2_ treatment. (TIF)

S2 Fig Extent of trans-activation of the *DkERF* promoters by DkTGA1, using the dual-luciferase assay. (TIF)

S3 Fig Yeast two-hybrid analysis of interaction of DkTGA1 and DkERF9. (TIF)

## Abbreviations

ADH: alcohol dehydrogenase
ERF: ethylene response factor
PDC: pyruvate decarboxylase
SCT: soluble condensed tannins
AD: pGADT7
BD: pGBKT7
AbA: aureobasidin A
cv: Cultivar
