# Supplementary figures and images for: Involvement of DkTGA1 Transcription Factor in Anaerobic Response Leading to Persimmon Fruit Postharvest De-Astringency
Source: PLoS One. 2016 May 19;11(5):e0155916. doi: 10.1371/journal.pone.0155916 (PMC4873192; doi:10.1371/journal.pone.0155916)

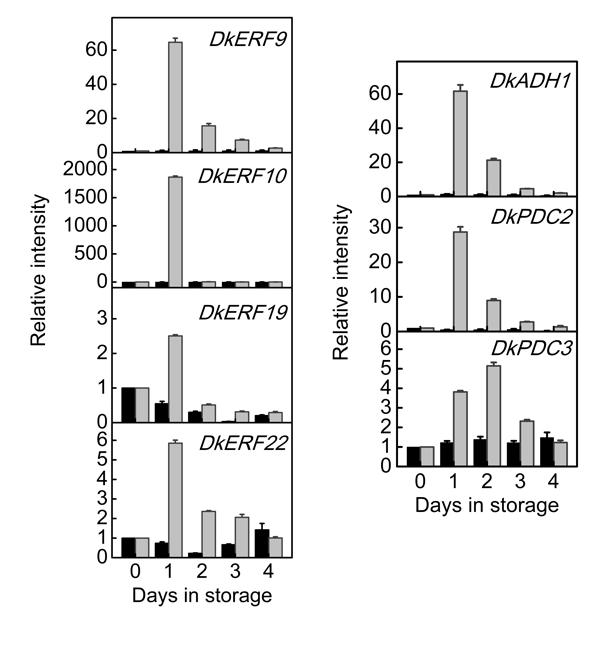

Supplement: S1 Fig — (TIF) [file pone.0155916.s001.tif]

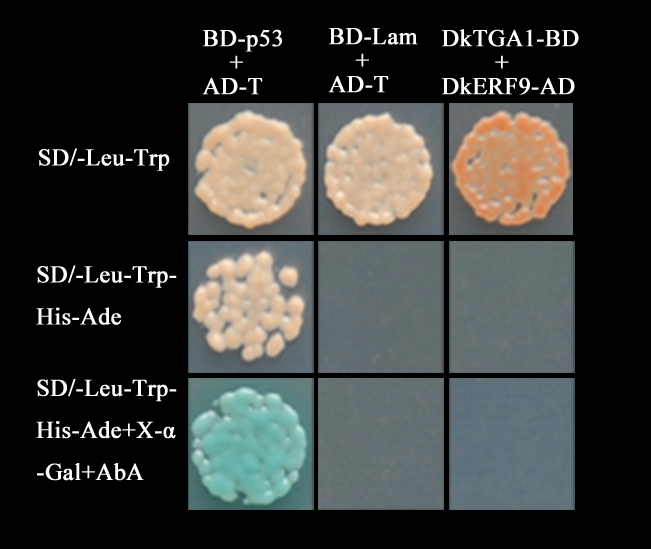

Supplement: S3 Fig — (TIF) [file pone.0155916.s003.tif]
